# Clinical Risk Factors Associated with Poor Outcomes in Snake Envenoming: A Narrative Review

**DOI:** 10.3390/toxins15120675

**Published:** 2023-11-28

**Authors:** Darryl Wood

**Affiliations:** 1 Department of Emergency Medicine, Blizzard Institute, Queen Mary University, London E1 2AT, UK; darrylrwood@yahoo.co.uk; 2 Queens Hospital, Barking, Havering and Redbridge University Trust, Rom Valley Way, Romford, London RM7 0AG, UK

**Keywords:** snakebite, envenoming, antivenom, pregnancy, children, acute kidney injury, haemorrhage

## Abstract

Snakebite-related fatalities disproportionately affect populations in impoverished socio-economic regions, marked by limited access to adequate healthcare and constrained antivenom availability. Early medical intervention is pivotal in mitigating mortality and morbidity associated with snakebite envenoming (SBE). While clinical assessment remains fundamental in treating SBE, this review aims to spotlight objective parameters that could also affect outcomes. Selected studies that identify factors associated with poor outcomes are predominantly region-specific, single-site, and observational, yet collectively reveal similar findings. They consistently report factors such as treatment delays, susceptibility in vulnerable groups such as children and pregnant women, as well as various biochemical and haematological abnormalities. Acute kidney injury (AKI), low platelets, leucocytosis, abnormal coagulation, and elevated creatine kinase (CK) all show an association with poor outcomes. Furthermore, recognising rare and unusual SBE presentations such as adrenal insufficiency, severe hypertension, intracranial haemorrhage, acute angle closure glaucoma, and bowel ischaemia also has a bearing on outcomes. Despite the integration of these parameters into clinical decision tools and guidelines, the validation of this evidence is limited. This review underscores the imperative for high-quality, multi-centre studies aligned with consensus-driven Core Outcome Sets (COS) and Patient-Reported Outcome Measures (PROMS) to validate and strengthen the current evidence.

## 1. Introduction

It is estimated that 2.7 million people suffer from snakebite envenoming (SBE) annually, resulting in approximately 81,000 to 138,000 deaths [1]. These figures, considered conservative due to underreporting and insufficient case coordination, underscore the significant global impact of SBE. In perspective, nearly 7400 individuals are bitten by snakes daily. The morbidity associated with SBE is challenging to quantify, prompting the World Health Organization (WHO) to target a 50% reduction in mortality and disability by 2030 through improved clinical decision-making, treatment, and recovery [1].

SBE predominantly affects tropical and subtropical regions in lower to middle-income countries, where poor and rural populations face limited access to healthcare and antivenom [2,3]. While mortality from SBE is relatively low, the associated morbidity is unknown and difficult to quantify. In many countries, snakebite is not reportable, and complications are often not attributed to SBE in official reports, indicating a gap in health data collection [2,3,4]. The WHO advocates for increased awareness and inclusion of snakebite in the portfolio of neglected tropical diseases [4].

Early antivenom treatment is crucial for reducing both mortality and morbidity [3,4,5]. Long term effects, such as cytotoxic tissue destruction leading to wound complications that may require skin grafting and even amputations, may necessitate extensive rehabilitation. Organ injuries, such as renal failure, may require resource-intensive renal replacement therapy or dialysis, significantly impacting healthcare and social support systems. Globally, morbidity consequences are conservatively estimated at 6.07 million disability-adjusted life years [3,6,7].

Frontline clinicians often lack experience, training, and support from experts in snakebites and toxinology [3,8]. Relying on local experience, guidelines, and online platforms such as Toxbase^®^ [9] in the United Kingdom, management decisions are inconsistent and lack robust scientific evidence. Current clinical evidence on SBE is of low quality, fragmented, and lacks coordination across regions [10]. Although evidence associating risk factors with outcomes exists, there is a scarcity of high-quality evidence for objective factors that predict unfavourable outcomes.

The cornerstone of SBE treatment is antivenom, but its administration demands careful consideration, especially in areas with limited supply [11]. The definition of a poor outcome varies, but end points such as mortality, hospital length of stay, ventilation, antivenom, and surgical procedures are consistently reported [12,13,14]. Clinicians must balance the benefits of antivenom against the risk of life-threatening anaphylaxis, which is more likely in the less purified, polyvalent antivenoms [3]. 

**Figure 1:**
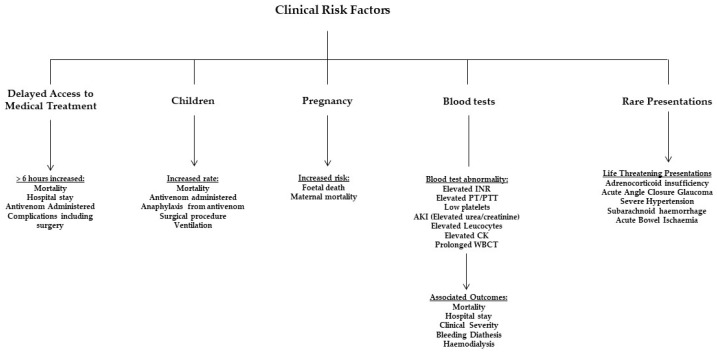
Summary of the clinical risk factors associated with poor outcomes.

Early treatment decisions are imperative for effective SBE management, and identifying outcome-affecting factors can expedite clinical decisions. Acknowledging the limitations of frontline staff who rely on guidelines and diagnostic algorithms is common during the critical early hours post-bite. However, assessing the effectiveness of these tools is challenging due to the lack of high-quality research. This review will evaluate the published evidence on some of the factors that independently and collectively pose a risk to SBE patients and explore their potential incorporation into clinical decision-making. Figure 1 summarises these risk factors and their associations with outcomes. 

Medical search heading terms used to source relevant papers included “snakebite”, “envenoming”, “antivenom”, “poor outcomes”, “adverse outcomes”, “children”, “paediatrics”, “pregnancy”, “delayed treatment”, “blood tests”, “haemotological tests”, “acute kidney injury”, renal failure”, “whole blood clotting time”, “snakebite score”, “snakebite scale”, “rare snakebite presentations”, “mortality”, “morbidity” and “snakebite research” using combination Boolean operator terms “and”/“or”. The databases searched included PubMed, Google Scholar, Cochrane collaboration, and Embase. Table 1 presents the selected studies that report factors that are associated with poor outcomes.

## 2. Delays to Treatment

Patients from rural communities who suffer snakebites often initially turn to traditional healers, primarily due to traditional beliefs and the significant distances to medical care facilities [27,28]. These factors can lead to time-sensitive delays in receiving prompt and definitive treatment. In some cases, patients may spend hours or even days before reaching a medical facility equipped to treat SBE. Such delays, especially in those patients with severe SBE, have been shown to increase the risk of a poor outcome [12,14,15,29,30,31,32].

Once SBE occurs, there is a race against the clock for patients to access healthcare and for clinicians to administer antivenom to neutralise venom toxins and prevent irreversible damage to tissues. Early antivenom serves as the definitive treatment for SBE and is effective at reducing both morbidity and mortality when administered promptly [3,5,6].

To maximise the efficacy of antivenom therapy, it is crucial for the antivenom to effectively bind to venom proteins before they disseminate to their intended target sites, where they can induce irreversible damage [32]. The timely administration of antivenom can yield significant benefits, including the mitigation of neurotoxic weakness, restoration of normal coagulation processes, and reduction of irreversible tissue damage caused by cytotoxins [3,32,33,34].

Neurotoxins, specifically, target both pre- and post-synaptic terminals at the neuromuscular junction (NMJ), leading to necrotic degeneration within the initial 12 h following envenomation [32]. Once neurotoxins bind to the NMJ, the effectiveness of antivenom in preserving neuromuscular function diminishes considerably. The recovery of nerve terminals can be a protracted process, requiring up to 7 days for the full restoration of neuromuscular function. Consequently, individuals who do not receive prompt antivenom treatment may necessitate mechanical ventilation for a period extending up to a week [32]. The effectiveness of antivenom is also time-sensitive in cases of venom-induced consumption coagulopathy (VICC). Ideally, antivenom needs to bind to procoagulant toxins before the clotting pathway is activated, in order to exert its therapeutic benefits [35]. Furthermore, cytotoxins, which are responsible for inducing irreversible necrosis and extensive tissue damage, can have their destructive tissue effects limited and even prevented when antivenom is administered early [3].

While under 6 h is loosely described as the treatment time target [15,31,36], this has not been clearly defined, with studies reporting different time frames. Jayaram et al. [16] report a “bite to needle” time of more than 6 h as having worse outcomes, such as longer hospital stays, more antivenom required, and higher complication rates. The mortality rate in patients who received antivenom for more than 24 h after the bite was high (8/12 patients). Another report from India showed that a delay of more than 24 h had an associated mortality of 18% versus 5% in patients who were hospitalised earlier [14].

In Brazil, investigators demonstrated that the rate of a bad outcome increased significantly with each time frame delay, from 3.6% (0–3 h), 5.5% (3–6 h), 6.5% (6–24 h), and 12.2% (>24 h) [17]. Reports from southern Africa have associated delays to definitive care of more than 7 h as an independent predictor for the need to prescribe antivenom or the need for a surgical procedure [15]. Collectively, these regional studies support the clinical opinion that delays in treatment render antivenom less effective and increase the likelihood of an unfavourable outcome. While the optimal and pragmatic timeframe for administering antivenom has not been established in clinical trials, it is intuitive that the earlier it is administered, the more effective it will be. In this regard, rapid clinical decision-making is imperative. Delays to definitive treatment appear to be a strong predictor of worse outcomes following SBE.

## 3. Paediatrics

Children pose unique clinical challenges to clinicians treating SBE. In rural areas, children have a heightened propensity for snake encounters, as evidenced by the prevalence of up to one-third of reported cases in Nigeria and Australia [18,19,37] and 15% of reported snakebite cases in Brazil [38]. The reasons are most likely due to children’s playing activities, inquisitive nature, and lack of appreciation for the health risk associated with a snakebite. Young children and toddlers often exhibit an unreliable recollection of the incident, lacking clarity regarding the timing and identification of the snake species involved [39,40]. These factors can hinder prompt medical interventions and delay the administration of essential antivenom therapy.

The clinical signs and symptoms following SBE are similar for children and adults [20]. However, the envenomation dose delivered by snakes is not discriminatory, exposing children to a larger venom dose-to-weight ratio than adults [11]. This has a direct impact on the clinical severity and complication risk for children [19,20,40,41].

One study from South Africa reported an odds ratio of 2.13 for children requiring antivenom or a surgical procedure, while another cited a relative risk of 4.75 for a surgical intervention when compared to adults [15,20]. Overall, young children and toddlers carry a higher risk of poor outcomes [13,38]. A study from Nigeria reported that children with cytotoxic SBE are more likely to need an amputation, blood transfusion, and antivenom than adults [19,40]. Antivenom is more commonly prescribed for children with snakebite than adults in sub-Saharan Africa, with the proportion ranging from 15–85% [13,15,18,19,20]. One study in India reported an odds ratio of 2.97 for a poor outcome (mortality, ventilation, or renal replacement therapy) in children under 5 years of age [13]. In this study, snakebite-related mortality was unusually high at 11.7%, while in South Africa, despite an increased risk of severity, the recorded mortality rates in children remained low, with few reported deaths [12,20,40].

The dose of antivenom in children and adults is equivalent since it depends on the snake species and the volume of venom delivered [11]. Dosing is not dependent on body weight but rather on the amount of antivenom that is required to neutralise circulating snake venom. This poses a clinical challenge when considering the volume of antivenom to be administered to babies or small toddlers. Some protocols for antivenom administration suggest diluting the antivenom in 200 mL of crystalloid fluid [42], which may result in volume overload in babies, especially if doses need to be repeated.

To add insult to injury, the rate of anaphylaxis from antivenom in children is higher than in adults. In East Africa, the rate of acute allergic reactions from several different antivenom products was reported at 17.6% in children [18]. The less-purified polyvalent antivenoms are more likely to cause allergic reactions and anaphylaxis than the more-purified or monovalent antivenom products. Southern Africa utilises a polyvalent antivenom, and reported rates of anaphylaxis in children vary between 25 and 57% [12,20,40]. Some of these cases developed anaphylactic shock that required cardio-pulmonary resuscitation and ICU admissions. 

Collectively, these observations underscore the heightened severity and distinctive clinical characteristics of SBE in paediatric patients, such as mortality, hospital admissions, the need for surgical procedures, and the risk of antivenom-associated anaphylaxis. The presented evidence associates the paediatric population as an independent predictor of an elevated risk of poor outcomes following SBE [15].

## 4. Pregnancy

Snakebite envenomation (SBE) can affect both the mother and foetus during pregnancy, leading to adverse outcomes such as maternal death, abruptio placenta, stillbirth, miscarriage, and preterm delivery [21,43]. Although the evidence is limited, these adverse outcomes appear to be primarily related to the effects of the venom on the mother [43]. One report from Brazil showed that envenomed pregnant women have a higher risk for foetal (Odds Ratio 2.17) or neonatal death (Odds Ratio 2.79) [19]. Foetal loss is more likely to occur early in the gestation period [21,44]. Abruptio placenta, especially in haemotoxic SBE, is the commonest cause of foetal loss [45]. Interestingly, the Brazilian study [21] showed no difference in severity or case fatality between pregnant and non-pregnant women of childbearing age. In contrast, earlier studies demonstrated a significant increase in maternal mortality in envenomed pregnant women. One review in 2010 reported a case fatality rate of 4.2% and 19.2% in pregnant women and neonates, respectively [22]. Mechanisms proposed to explain foetal deaths included anoxia secondary to maternal shock, abruptio placenta, venom-induced premature uterine contractions, and a direct venom effect on the foetus [45].

Antivenom is still considered the definitive treatment in pregnancy with the same indications. However, the safety of antivenom in pregnancy has not been evaluated in robust clinical trials [44]. Although evidence-based recommendations are limited, the management of SBE in pregnancy includes supportive care, administering antivenom in severe cases, anticipating and promptly treating anaphylaxis, and close maternal-foetal monitoring during the admission [43].

## 5. Blood Parameters

Blood tests are a useful tool when managing snakebites. Venom toxins can have systemic effects that affect several blood parameters, which can be measured. Baseline blood tests usually include a 20-min whole blood clotting time (20WBCT), a full blood count, urea and creatinine (kidney function) levels, coagulation parameters, and creatine kinase [34]. At a minimum, these tests can give clinicians a baseline profile of a patient’s blood composition and highlight any abnormalities. 

Several studies have associated abnormal blood results with unfavourable outcomes [15,23]. In South Africa authors presented the Zululand Snakebite Score, which identified some blood parameters that were predictors of an unfavourable outcome. Of note were a raised INR (>1.2), low platelets (<92 × 10^9^/L) and a low haemoglobin (<7.1 g/dL) [15]. These were combined with other parameters into a severity scoring system. Low platelets (<60,000), acute kidney injury indicated by abnormal urea and creatinine levels, and coagulation derangement (PT > 13.2 s, PTT > 37.2 s) were associated with a worse outcome in southern India [14]. Ozbulat et al. showed that low haemoglobin, low platelets, and elevated creatinine kinase (CK) were correlated with longer hospital stays [23]. Authors in north Africa reported elevated leucocytes and elevated CK levels as independent predictors of severity, while low platelets were associated with more severe presentations [24]. In sub–Saharan Africa, low haemoglobin, raised leucocytes, and low serum sodium were independently associated with severity in envenomed children [18]. Although some explanations for abnormal blood results are speculative, some mechanisms have been proposed. A bleeding diathesis is associated with abnormal coagulation parameters such as PTT, aPTT, INR, and fibrinogen levels, which are indicative of a venom induced consumption coagulopathy (VICC) and haemorrhage [34]. Low platelets may contribute to the bleeding diathesis because of microangiopathic haemolysis or transient consumptive thrombocytopenia. Leucocytosis suggests a systemic inflammatory response to the snake venom, while elevated CK levels (>10,000 U/L) are indicative of a significant rhabdomyolysis potentially causing an acute kidney injury (AKI) [34].

Abnormal urea and creatinine levels can alert the clinician that the patient is developing an AKI that may progress and require renal replacement therapy. Scattered studies have cited a wide range (8–50%) of snakebite-related AKI cases that will progress to chronic kidney disease (CKD) [46]. Primvada et al. noted that patients with a significant AKI (KDIGO stage 3) were associated with higher mortality [25]. They also reported that one-third of patients with a snakebite-associated AKI will develop CKD in the long term.

Another simple, front-line test is the 20 min whole blood clotting time (20WBCT) that has been used to assess SBE-related coagulopathy for decades. It is a bedside test that is cheap, easy to perform, and provides clinicians with an early diagnosis of VICC. A few millilitres of blood are added to a glass tube and left for 20 min. Blood that fails to clot after this period is indicative of a coagulopathy. The 20WBCT is a highly specific and reasonably sensitive test in the context of severe cases where there is an established VICC. A sensitivity of 82–89% and a specificity of 82–98% have been reported in clinical validation studies on 20WBCT [26]. However, one report suggested that the test might potentially miss one of every five coagulopathic patients [47]. Findings from a recent systematic review and meta-analysis of the 20WBCT indicated an 84% sensitivity and 91% specificity using the international normalised ratio (INR > 1.4) and a 72% sensitivity and 94% specificity using plasma fibrinogen (fibrinogen < 100 mg/dL) [26]. The likelihood of a bleeding diathesis and a poor outcome is higher in these cases. However, it has been shown that the WBCT test is less accurate in mild or early venom-induced coagulopathy [26,48].

The utility of bedside thromboelastography (TEG) is well-recognised in the context of trauma, and its potential application in the setting of SBE has been proposed [49,50,51]. TEG enables rapid interpretation of the blood’s clotting status and provides the capability to distinguish between various variables that impact coagulation, such as platelets, clotting factors, and fibrinogen [50,51]. TEG represents a dynamic mechanical assessment of the coagulation process, encompassing the stages from fibrin formation to platelet aggregation and fibrinolysis [49]. TEG can be used as an additional point-of-care test to give clinicians real-time information on coagulation and to promptly respond to any detected abnormalities [50]. The prohibitive costs and technical challenges associated with its implementation may hinder its broader utilisation, particularly in under-resourced healthcare facilities [49]. However, the association between TEG and clinical outcomes remains to be firmly established, necessitating further well-designed studies to clarify this relationship.

These observations highlight the critical role of early phlebotomy and blood testing for essential biochemical and haematological data in order to identify objective abnormalities that may predict patient outcomes. Simple point-of-care tests such as the WBCT and TEG can fast-track clinical decision-making. Clinicians can leverage this knowledge to make informed decisions and initiate timely antivenom administration in SBE.

## 6. Scoring Tools

While clinical evaluation remains fundamental in snakebite envenomation (SBE), the integration of objective parameters that impact patient outcomes into scoring systems may enhance diagnostic objectivity and aid clinical decision-making [15,52,53]. Several scoring systems have been developed to risk-stratify patients with SBE.

A simple scale used in Martinique [54] to grade patients bitten by the lancehead species (*Bothrops lanceolatus*) focuses on signs and symptoms of swelling, pain, and systemic manifestations. Minor presentations on this scale are given a score of 1, while severe swelling with systemic features is assigned a score of 4. Clinicians utilise this grading scale to guide the dose of antivenom required [54].

The Snakebite Severity Score (SSS), proposed in North America for crotalid bites, is a detailed scoring system (Table 2) that provides a comprehensive framework based on clinical assessment and coagulation tests to facilitate risk stratification of SBE [53]. The score ranges from 0 to 20, with higher scores indicating greater severity. However, it is limited by a reliance on subjective judgment by the physician, the time it takes to complete the score in the clinical setting, and the fact that it has been validated retrospectively.

Meanwhile, the simplified version of the SSS, introduced by Sharman et al. [55], is exclusively a clinical score. Although it offers a practical alternative with reduced complexity, haematological and demographic parameters are not included.

A mortality risk prediction score called the Venom score (Table 3) for Russell’s viper (*Daboia russelii*) envenoming in southern India included seven admission parameters associated with mortality [52]. While haemoglobin was included in the score, other blood parameters such as prothrombin time (PT), activated partial prothromboplastin time (aPTT), serum fibrinogen, and D-dimers were not included for pragmatic reasons such as the limited supply and shortage of these tests in primary care settings. However, parameters such as time to antivenom administration and gender were included. The authors report that a score > 6 carries a significant risk for mortality, and patients may require early intensive care [52]. The predicted mortality rate for a score of 7 is 22%, and the maximum score of 12 is 99.1%.

In southern Africa, the Zululand Snakebite Score (ZSS) adopts a multi-dimensional approach. It utilises population demographics, timing of treatment, haematological, and coagulation parameters to risk-stratify snakebite envenoming (Table 4) [15]. The ZSS describes a score of ≥4 predictive of an active treatment intervention (ATI), defined as the need for a surgical procedure in severe cytotoxic tissue damage or the need to administer antivenom. The ZSS is incorporated into treatment guidelines that include clinical parameters.

Such scoring systems are an attempt to facilitate clinical decisions using a number of different parameters, but they require rigorous validation to ascertain their reliability and clinical utility.

## 7. Rare Presentations and Poor Outcomes

To effectively manage snakebite incidents, it is imperative to provide comprehensive training to frontline clinical personnel, enabling them to adeptly address major snakebite presentations [56]. Proficiency in recognizing the predominant clinical signs and symptoms of SBE is pivotal for ensuring timely and appropriate interventions [56,57]. However, the existence of infrequent and uncommon SBE manifestations poses a significant challenge, potentially leading to adverse patient outcomes. A number of rare SBE presentations have been reported in the literature, and the clinical recognition of these signs has a bearing on patient outcomes.

Endocrine manifestations, though rare, can carry life-threatening consequences. Venom-induced adrenal crisis, observed particularly in Russell’s viper (*Daboia russelii*) envenoming [57,58] is a severe manifestation. The mechanism is thought to be due to venom-induced haemorrhages in the pituitary or adrenal glands, which result in hypotension and circulatory collapse [34,58]. A case from India [57] reported a patient bitten by a Russell’s viper who had persistent shock, despite antivenom treatment followed by adrenaline and dexamethasone in anticipation of anaphylaxis. Laboratory tests showed hyponatraemia, hyperkalaemia, hypoglycaemia, and reduced cortisol production. CT scanning demonstrated bilateral haemorrhages in the pituitary and adrenal glands. The patient was further managed with hydrocortisone and thyroxine and made a good recovery [57]. In such cases, the timely recognition of the clinical presentation and the administration of glucocorticoids are critical.

The typical ocular manifestations resulting from SBE include direct injury to the cornea from venom sprayed into the eyes by species such as the spitting cobras or ophthalmoplegia due to neurotoxicity and ocular muscle paralysis [59,60]. The presence of ptosis following Crotalus neurotoxin envenoming in Brazil has been associated with severity and mortality (likelihood ratios of 1.4 and 3.8) [61]. Less common ocular manifestations can occur with viper bites, such as acute angle-closure glaucoma. It is thought to be caused by venom-induced ocular capillary leak syndrome, which causes increased pressure in the anterior chamber of the eye and may lead to blindness if left untreated [59,60]. Other rare presentations include optic neuritis and vitreous haemorrhages. Early detection and appropriate management of ocular sequelae can prevent visual loss in affected patients [60].

Severe hypertension has been reported following elapid envenoming. In one case series, systolic blood pressures in excess of 200 mmHg and diastolic blood pressures of between 120 and 180 mmHg were documented in patients envenomed by the Indian krait (*Bungarus caeruleus*) [62]. Autonomic dysfunction, possibly linked to neurotoxin inhibition of the presynaptic alpha-2 adrenoreceptors, affecting the release of norepinephrine, is postulated as the underlying mechanism [62]. Patients in this series required antivenom and ventilatory support. Despite this, their hypertension persisted and required further management with intravenous nitroglycerine. All the patients recovered after a few days with good outcomes.

Cerebral complications from SBE are not common but can occur across a number of species. Ischaemic stroke and intracerebral haemorrhage are the most common cerebral complications reported in viper bites [63]. Viper venom contains a number of proteins that have procoagulant effects, resulting in an increased risk of bleeding. Conversely, viper venom proteins may also have prothrombotic effects, causing platelet aggregation and ischaemic stroke [63,64]. One particularly rare but potentially fatal cerebral presentation is subarachnoid haemorrhage (SAH). One case reported a patient who was poorly responsive with a depressed level of consciousness following Russell’s viper envenomation. Significant features in the diagnostic workup showed a prolonged WBCT and a CT scan with evidence of a SAH [64]. The management included antivenom for the envenoming and treatment for the SAH with mannitol and nimodipine in ICU. Following a prolonged hospital stay, the patient was eventually discharged with outpatient follow-up as her symptoms had improved.

SBE may manifest with abdominal complications, often heralded by severe abdominal pain and discomfort. These symptoms are often mistaken for other abdominal pathologies, such as appendicitis, peritonitis, or cholecystitis [65]. A case following lancehead (*Bothrops atrox*) envenoming reported a patient with severe abdominal pain in the right flank and iliac fossa [65]. The cause of the abdominal pain was identified as acute mesenteric and bowel ischaemia, as evidenced on CT scan. Although the mechanism is not fully understood, the prothrombotic effects of venom proteins are thought to increase platelet activity through the formation of distal microthrombi and visceral infarctions [66]. Although the patient had antivenom administered, surgery was performed, and the patient made a full recovery [65].

Another unusual abdominal presentation following SBE is rectus abdominal sheath haematoma. There are rare reports of rectus sheath haematomas following Russell’s viper bites [67]. This is caused by bleeding and haematoma formation in the rectus abdominus sheath. Acute presentations manifest with severe localised abdominal pain with nausea, vomiting, and rigors. Appropriate treatment includes administering antivenom, blood products, analgesia, and a period of observation. In severe cases where abdominal compartment syndrome is present, surgery may be indicated [68].

Recognition of these and other unusual clinical presentations is crucial for expediting appropriate treatment, which can be sight-, limb-, and life-saving. However, mitigating against these rare presentations is a challenge for most frontline clinicians. Table 5 summarises these rare SBE presentations and their potential adverse outcomes.

## 8. Looking to Future Research

The WHO’s target of reducing SBE deaths by 50% by 2030 underscores the urgent need to direct research efforts towards clinically relevant studies that identify and validate risk factors predictive of poor outcomes [1,69]. One of the key pillars of the WHO’s strategy to reduce mortality is ensuring safe and effective treatment [1]. Strategies targeting risk factors for poor patient outcomes would contribute to better treatment options for SBE. However, the quality of the evidence informing clinicians on the best practices for managing SBE is insufficient. Furthermore, regions such as sub-Saharan Africa and the Middle East have fallen short in delivering adequate research on SBE.

The prevailing body of clinical evidence largely comprises isolated observational studies characterised by inadequate statistical power and, notably, the infrequent performance of power calculations. The availability of rigorous clinical trials within the domain of clinical snakebite research remains conspicuously scarce. One salient factor contributing to this scarcity is the dearth of financial resources allocated to support extensive multicentre trials. In 2017, the World Health Organization elevated snakebite envenoming as a category A neglected tropical disease [1,11,70], providing opportunities for accessing funding for SBE research projects. However, consensus on defining appropriate PICO (population, intervention, comparator, outcome) parameters that are relevant to patient outcomes is key to validating associated surrogate endpoints and informing health authorities.

Outcome measures vary across studies and are often not designed to validate identified surrogate endpoints. One initiative is the Core Outcome Measures in Effectiveness Trials (COMET), which aims to identify Core Outcome Sets (COS) in SBE [70]. This will allow researchers to define important Patient-Related Outcome Measures (PROMs) and design studies in line with a coordinated framework. By reducing heterogeneity in the studies’ PICO framework, researchers would be able to compare, contrast, and combine the results of clinical trials more accurately, especially when conducting systematic reviews and meta-analyses. PROMs would need to consider risk factors that are predictors of poor outcomes, such as delayed treatment, vulnerable groups such as children and pregnant women, and blood parameters such as coagulation and renal function tests. Quantifying and validating these factors will allow for a more strategic approach to reducing risks for patients experiencing unfavourable outcomes in SBE.

## 9. Conclusions

The timely administration of antivenom is pivotal in averting adverse outcomes associated with snakebite envenomation (SBE). In regions with limited antivenom supply, treatment is often reserved for those at the highest risk, emphasising the need for parameters predicting unfavourable outcomes in SBE. Studies identifying these factors are generally region-specific, single-site, and observational in design. However, they cumulatively report similar findings despite regional variations in snakebite presentations. Clinicians should be cognisant of rare SBE presentations that have a bearing on adverse outcomes. Developing and validating scoring systems that include factors predictive of poor outcomes aims to strengthen clinical decision-making. Strategic interventions targeting these parameters, such as reducing treatment delays and enhancing antivenom access for vulnerable groups, can profoundly impact patient outcomes.

Collaborative efforts using appropriate Core Outcome Sets (COS) and Patient-Related Outcome Measures (PROMs) in larger, multi-centre studies are warranted to bolster the evidence base, ultimately refining treatment strategies and improving patient outcomes in SBE.

## Figures and Tables

**Table 1:** Studies that highlight SBE-related risk factors that are associated with unfavourable patient outcomes.

Risk Factor	Type of Study	Country	Main Result	Outcome	Reference
Delayed Treatment	Retrospective Observational	India	Increased mortality; 18% > 24 h delay vs. 5% < 24 h delay	Mortality	Suresh et al. [14]
	Validation Cohort Observational	South Africa	Delay > 7 h is an independent predictor of a poor outcome	The need for an ATI (antivenom or surgery)	Wood et al. [15]
	Prospective Observational	India	“Bite to Needle” time > 6 h had poor outcomes	Longer hospital stay More antivenom administered Higher complication rates High mortality > 24 h (90%)	Jayaram et al. [16]
	Retrospective epidemiological study	Brazil	6.5% (>6 h delay) and 12.2% (>24 h delay) of patients had a poor outcome	Mortality Local complication (secondary infection, necrosis, amputation) Systemic complication (AKI, shock, sepsis)	Schneider et al. [17]
Children	Retrospective Cohort Observational	India	Children < 5 years; OR 2.97 for a poor outcome; 11.7% mortality	Mortality Ventilation Renal replacement therapy	Suryanarayana et al. [13]
	Validation cohort Observational	South Africa	Age < 14 years; OR 2.13 for a poor outcome	The need for an ATI (antivenom or surgery)	Wood et al. [15]
	Prospective and Retrospective Observational	Kenya	Acute allergic reaction occurred in 17.6% of a number of different antivenom products	Anaphylaxis	Abouyannis et al. [18]
	Prospective Observational	Nigeria	85% in children vs 80% in adults (*p* < 0.05) had poor outcomes	Antivenom Amputations Blood transfusion	Iliyasu et al. [19]
	Retrospective Observational	South Africa	Age < 13 years; RR 4.75 for poor outcome compared to adults	Surgical procedure, e.g., debridement, skin graft, amputation	Buitendach et al. [20]
Pregnancy	Retrospective Cohort	Brazil	Pregnant women: OR 2.17 for foetal death and an OR 2.79 for neonatal death	Mortality	Nascimento et al. [21]
	Literature review	Global	Case fatality rates of 4.2% in pregnant women and 19.2% in neonates	Mortality	Langley et al. [22]
Blood Tests	Retrospective Observational	India	Platelets (<60,000) and coagulation abnormalities (PT > 13.2 s, PTT > 37.2 s) are associated with mortality. AKI: 21% mortality vs. 3% without AKI; 15.3% required haemodialysis	Mortality Haemodialysis	Suresh et al. [14]
	Validation cohort Observational	South Africa	INR (>1.2), low platelets (<92 × 10^9^/L), and low haemoglobin (<7.1 g/dl) predict a poor outcome	The need for an ATI (antivenom or surgery)	Wood et al. [15]
	Observational	Turkey	Increased hospital atay: Hb < 11.6 g/dl; Platelets < 156 × 10^3^/µL; CK > 234 U/L	Length of hospital stay	Ozbulat et al. [23]
	Retrospective Observational	Tunisia	Independent predictors for severe SBE: leucocyte > 11 550/mm3 (OR 18) and CK > 155 IU/L (OR 6.16)	Severe SBE *	Chakroun-Walha O [24]
	Prospective Observational	India	AKI stage 3: RR 4.45 for death	Mortality	Priyamvada et al. [25]
	Systematic Review	Global	WBCT test for coagulopathy: INR > 1.4 = 84% sensitivity and 91% specificity Fibrinogen < 100 mg/dl = 72% sensitivity and 94% specificity	Bleeding	Lamb et al. [26]

Hrs = Hours; AKI = Acute Kidney Injury; ATI = Active Treatment Intervention; OR = Odds Ratio; RR = Relative Risk; Hb = Haemoglobin; CK = Creatinine Kinase; SBE = Snakebite Envenoming; WBCT = Whole Blood Clotting Time. * Severe SBE reported as = Hematologic abnormalities, Haemorrhage, Organ failure (respiratory failure, shock, neurological abnormalities), Limb necrosis, compartment syndrome or impending compartment syndrome requiring emergency fasciotomy, mortality.

**Table 2:** Snakebite severity score for North American crotalid bites [52].

Parameter	Score
**Pulmonary symptoms** • No signs/symptoms • Dyspnea, minimal chest tightness, mild/vague discomfort, respirations of 20–25 bpm • Moderate respiratory distress, 26–40 bpm • Cyanosis, air hunger, extreme tachypnea, or respiratory insufficiency/failure	0 1 2 3
**Cardiovascular system** • No signs/symptoms • HR 100–125 BPM, palpitations, generalized weakness, benign dysrhythmia, or hypotension • HR 126–175 BPM, or hypotension with SBP >100 mmHg • HR >175 BPM, or hypotension with SBP <100 mmHg, malignant dysrhythmia, or cardiac arrest	0 1 2 3
**Gastrointestinal system** • No signs/symptoms • Pain, tenesmus, or nausea • Vomiting or diarrhea • Repeated vomiting, diarrhea, hematemesis, or hematocheszi	0 1 2 3 4
**Hematologic symptoms** • No signs/symptoms • Coagulation parameters slightly abnormal: PT < 20 secs, PTT < 50 secs, platelets 100–150K/mL, or fibrinogen 100–150 mcg/mL • Coagulation parameters abnormal: PT < 20–25 secs, PTT < 50–75 secs, platelets 50–100K/mL, or fibrinogen 50–100 mcg/mL • Coagulation parameters abnormal: PT < 50–100 secs, PTT < 75–100 secs, platelets 20–50K/mL, or fibrinogen <50 mcg/mL • Coagulation parameters markedly abnormal, with serious bleeding or the threat of spontaneous bleeding; unmeasureable PT	0 1 2 3 4
**Central nervous system** • No signs/symptoms • Minimal apprehension, headache, weakness, dizziness, chills, or parasthesia • Moderate apprehension, headache, weakness, dizziness, chills, parathesias, confusion, or fasciculation in area of bite site • Severe confusion, lethargy, seizures, coma, psychosis, or generalized fasciculation	0 1 2 3

**Table 3:** The Venom Score for mortality prediction in southern Indian viper bites [52].

Parameter	Score
**Gender** Female Male	1 0
**CLS** Yes No	2 0
**Bite to ASV time > 6.5 h** Yes No	1 0
**Bleeding** Yes No	3 0
**Haemoglobin** >10 g/dL <10 g/dL	1 0
**Urine output (in first 24 h)** <20 mL/hr >20 mL/hr	2 0
**Systolic BP** <100 mm Hg >100 mm Hg	2 0

CLS = Capillary Leak Syndrome.

**Table 4:** The Zululand Snakebite Score lists predictive factors for a poor outcome [15].

Active Treatment Intervention Risk Predictors	Allocated Score
Children < 14 years	1
Duration > 7 h	1
White Cell Count > 10 × 10^9^/L	1
INR > 1.2	1
Platelets < 92 × 10^9^/L	1
Haemoglobin < 7.4 g/dL	1

**Table 5:** Summary of rare snakebite cases associated with unfavourable outcomes.

Clinical Manifestation	Snake Species	Potential Outcome	Reference
Adrenocorticoid deficiency: hyperkalaemia, hypoglycaemia, hyponatraemia, hypotension	Russell’s viper (*Daboia russelii*)	Circulatory collapse and shock	Subramanian et al. [57]
Acute angle closure glaucoma with painful red eye and visual disturbance	Russell’s viper (*Daboia russelii*)	Increased pressure in the anterior chamber of the eye and the risk of blindness	Aye et al. [59] Kumar et al. [66]
Severe hypertension	Indian krait (*Bungarus caeruleus*)	Hypertensive emergency	Meenakshisundaram et al. [62]
Depressed level of consciousness, severe headache	Russell’s viper (*Daboia russelii*)	Sub-arachnoid haemorrhage	Roy et al. [64]
Diffuse abdominal pain	Lancehead (*Bothrops atrox*)	Bowel ischaemia	Galan et al. [65]
Focal abdominal pain	Russell’s viper (*Daboia russelii*)	Rectus abdominus sheath haematoma	Subramanian et al. [67]

## Data Availability

No original research data.

## Abbreviations

AKI	Acute Kidney Injury
CK	Creatine Kinase
CPR	Cardiopulmonary Resuscitation
COMET	Core Outcome Measures in Effectiveness Trials
COS	Core Outcome Sets
ICU	Intensive Care Unit
NMJ	Neuromuscular Junction
OR	Odds Ratio
PT	Prothrombin Time
PTT	Partial Thromboplastin Time
PICO	Population, Intervention, Comparator, Outcome
PROMS	Patient-Related Outcome Measures
RR	Relative Risk
SBE	Snakebite Envenoming
SSS	Snakebite Severity Score
TEG	Thromboelastography
VICC	Venom-Induced Consumption Coagulopathy
WBCT	Whole Blood Clotting Time
WHO	World Health Organisation
ZSS	Zululand Snakebite Score
